# Delivery of nanoparticles to brain metastases of breast cancer using a cellular Trojan horse

**DOI:** 10.1007/s12645-012-0029-9

**Published:** 2012-07-20

**Authors:** Mi-Ran Choi, Rizia Bardhan, Katie J. Stanton-Maxey, Sunil Badve, Harikrishna Nakshatri, Keith M. Stantz, Ning Cao, Naomi J. Halas, Susan E. Clare

**Affiliations:** 1 Department of Surgery, Indiana University School of Medicine, Indianapolis, IN 46202 USA; 2 Department of Chemistry, Rice University, Houston, TX 77005 USA; 3 Department of Pathology and Laboratory Medicine, Indiana University School of Medicine, Indianapolis, IN 46202 USA; 4 Laboratory for Nanophotonics, Rice University, Houston, TX 77005 USA; 5 School of Health Sciences, Purdue University, West Lafayette, IN 47907 USA; 6 Department of Radiology and Imaging Sciences, Indiana University School of Medicine, Indianapolis, IN 46202 USA

**Keywords:** Breast cancer, Brain metastasis, Gold-silica nanoshell, Nanoparticle, Blood–brain barrier

## Abstract

As systemic cancer therapies improve and are able to control metastatic disease outside the central nervous system, the brain is increasingly the first site of relapse. The blood–brain barrier (BBB) represents a major challenge to the delivery of therapeutics to the brain. Macrophages originating from circulating monocytes are able to infiltrate brain metastases while the BBB is intact. Here, we show that this ability can be exploited to deliver both diagnostic and therapeutic nanoparticles specifically to experimental brain metastases of breast cancer.

## Introduction

Brain metastases are a significant clinical challenge. They are diagnosed in 100,000–170,000 patients/year in the United States (Posner 1992; Weil et al. 2005) and outnumber primary brain tumors by a ratio of 10 to 1 (Cairncross and Posner 1983; Walker et al. 1985; Posner 1992). They are estimated to occur in 20–40 % of all cancer patients (Cairncross and Posner 1983; Posner 1995). The incidence rate of brain metastasis is thought to be increasing as a function of the aging population, better treatment of non-central nervous system (CNS) disease and improved imaging techniques. The most common primary tumor sites are lung (40–50 %), breast (13–17 %), melanoma (11–17 %), renal (6–16 %), and GI tract (4–6 %) (Barnholtz-Sloan et al. 2004; Klos and O’Neill 2004). At least 10 % of patients with primary small cell lung cancer (SCLC) have brain metastasis at diagnosis (Hochstenbag et al. 2000; Grossi et al. 2001), however a more recent prospective study of 432 patients in the Netherlands found the rate to be 18 % (Seute et al. 2004). Patients with locally advanced non-small cell lung cancer are at the highest risk for brain metastasis, with some studies reporting greater than 50 % of patients developing brain metastasis over the course of the disease (Stuschke et al. 1999).

Breast cancer metastatic to the brain is most prevalent in the triple negative (Lin et al. 2008) and HER2+ subpopulations (Bendell et al. 2003; Clayton et al. 2004; Lin and Winer 2007; Stemmler et al. 2006; Yau et al. 2006), where the median survival time ranges from 2 to 16 months and the mean 1-year survival is ~20 % (Mahmoud-Ahmed AS 2002). Since targeted therapies against HER2+ disease are able to successfully control non-CNS disease, the brain is increasingly being seen as the first site of relapse (Burstein et al. 2005). Currently, the mainstays of treatment for metastatic brain tumors are whole brain radiation therapy (WBRT), surgery, stereotactic radiosurgery or a combination of these modalities. However, significant neurotoxicity has been reported with the use of WBRT, resulting in endocrine dysfunction, significant memory loss and dementia (Sneed et al. 1999; Patchell and Regine 2003; Lo et al. 2005).

Drug uptake into the brain is limited by numerous factors, including physical barriers such as the blood–brain barrier (BBB) and the blood–cerebrospinal fluid (blood–CSF) barrier, and a substrate’s affinity for specific transport systems located at both of these interfaces (Graff and Pollack 2004; Pardridge 2005). Despite recent indications that chemical modulation of the BBB may be feasible (Carman et al. 2011), under physiological conditions, drugs and other substances can enter the brain only by passive transcellular diffusion, receptor-mediated transcytosis, or through the action of specific carrier systems. While in general, the more lipid-soluble a molecule is, the more readily it will penetrate the BBB and blood–CSF barrier to reach targets in the CNS, many lipid-soluble therapeutics have much lower brain permeability than would be predicted on the basis of their solubility (Begley 2004; Muldoon et al. 2007). In an experimental model of brain metastases of breast cancer, cytotoxic concentrations of paclitaxel were reached only in a subset (<10 %) of the “leakiest” metastases (Lockman et al. 2010), and this therapy was unable to reduce the metastatic burden in the brain over the period of the study. All of these factors render the brain a “sanctuary” site for metastases.

More than two decades ago, Fidler and colleagues provided evidence that macrophages of blood monocyte origin can infiltrate experimental brain metastases while the blood–brain barrier is intact (Schackert et al. 1988). Even earlier, Morantz and colleagues had quantified the content of macrophages in clinical specimens (Morantz et al. 1979). They examined 12 metastases to the brain from a variety of primary tumors. The mean content of macrophages was 24 % with a range of 4 to 70 %. The specific means by which macrophages are recruited to the metastasis is largely unknown. Primary brain tumors such as gliomas produce MCP1 and HGF/SF1, which attract monocytes (Strik et al. 2004). Whether there are similar immunomodulatory cytokines and chemokines elaborated by intracranial metastases has been “poorly investigated to date” (Strik et al. 2004).

The use of monocyte/macrophages as delivery vehicles to the CNS has been investigated in situations other than malignancy. Afergan et al. demonstrated the delivery of serotonin to the brain by monocytes, which had phagocytosed nano-liposomes containing this otherwise brain impermeant drug (Afergan et al. 2008). Dou and colleagues utilized bone marrow derived macrophages as carriers of and depots for antiretroviral drugs to treat and attenuate the symptoms of HIV-associated neurocognitive disorder (Dou et al. 2009). Therefore, we hypothesized that nanoparticle-laden monocytes/macrophages would home in to intracranial metastatic deposits by crossing the blood–brain barrier following injection into the systemic circulation. If successful, this would open the door to the use of monocytes/macrophages for the delivery of therapeutic nanovectors as well as nanoformulated therapeutics to malignant intracranial lesions.

## Materials and methods

### Fabrication of gold (Au) nanoshells

Au nanoshells (NS) [r_1_, r_2_] = [66, 80] nm were fabricated by seed mediated electroless plating of Au onto silica spheres as previously reported (Oldenburg et al. 1998; Brinson et al. 2008). Briefly, monodisperse silica nanospheres of 66 ± 2 nm radii were synthesized by the hydrolysis of tetraethylorthosilicate (TEOS) in basic solution via the Stoeber method (Stoeber et al. 1968). The silica particles are redispersed in ethanol and functionalized with (3-aminopropyl) triethoxysilane (APTES, Sigma) overnight. These amine-terminated silica nanospheres were washed twice to remove excess amines and redispersed in ethanol. The precursor nanoparticles were prepared by decorating the silica particles with small gold colloid (2–3 nm) fabricated by the method reported by Duff et al. (1993). The precursor particles were left unperturbed for 24 h at room temperature, following which they were washed and redispersed in H_2_O (10 mL). A plating solution was prepared in an amber bottle by mixing potassium carbonate (anhydrous, Fisher, Fairlawn, NJ) with 1 % HAuCl_4_ solution in H_2_O (200 mL). The plating solution was aged for 48 h before using it to fabricate the nanoshells. A continuous gold shell was grown around the silica nanospheres by mixing the plating solution with different aliquots of the precursor particles and bubbling CO_(g)_ for 10 s (Brinson et al. 2008). The reaction was scaled up to obtain the appropriate volume of nanoshells; the nanoshells were washed twice and finally redispersed in H_2_O.

### Isolation of Monocytes from Buffy Coat

Human whole blood was obtained from the Indiana Blood Center (Indianapolis, IN) for the isolation of monocytes. Lymphocytes, monocytes, and platelets, i.e., the buffy coat, were isolated using Ficoll (GE Healthcare Biosciences, Piscataway, NJ) density gradient centrifugation. Monocytes were separated from the other components of the buffy coat using MicroBeads conjugated to monoclonal mouse anti-human CD14 antibodies and MACS^®^ cell separation system (Miltenyi Biotec, Auburn, CA).

### Imaging of gold-silica nanoshells loaded in monocytes/macrophages

Five thousand monocytes/macrophages were incubated in 200 μl RPMI media supplemented with M-CSF with 8.3 × 10^8^ nanoshells for 3 days at 37 °C, 5 % CO_2_. The visualization was performed using a Bio-Rad Radiance 2100 MP Rainbow Confocal/Multiphoton System. The images were acquired using the ×10 objective.

### Imaging of red fluorescence microspheres loaded in monocytes/macrophages

Specimens for confocal fluorescence microscopy were prepared by pipetting 50 μL of the red fluorescently labeled microsphere suspension (FluoSpheres® NeutrAvidin® labeled microspheres; 0.04 μm; red fluorescent, excitation 580 nm, emission 605 nm; Molecular Probes, Invitrogen, Carlsbad, CA) onto the glass bottom of 35 mm glass bottom dishes (MatTek Corporation, Ashland, MA). The dishes were left at room temperature for 30 min to allow time for the particles to settle. Three thousand macrophages suspended in 100 μL of RPMI media were added to the glass bottom and the dishes were incubated at 37 °C for 1 h to allow the cells to attach. Three milliliters of RPMI supplemented with M-CSF was added to the cells and they were incubated for 3 days at 37 °C, 5 % CO_2_.

### Imaging of gold nanospheres and red fluorescence microspheres loaded in monocytes/macrophages

Electron microscopy specimens were prepared by adding 50 μL of the red fluorescently labeled microsphere suspension to a Thermanox coverslip [cut in half], which was placed in a 35 mm tissue culture dish. 50 μL of gold streptavidin conjugate, 20 nm were added to the same coverslip followed by 300,000 macrophages in 100 μL of media. Dishes were incubated at 37 °C for 1 h in order for the cells to attach. Three milliliters of RPMI supplemented with M-CSF was added to the dish and the dishes were incubated for 3 days at 37 °C, 5 % CO_2._ Cells were then fixed in 2 % paraformaldehyde, 2 % glutaraldehyde in 0.1 M PO_4_ buffer.

### Establishment of the brain metastatic tumor xenografts

The human MDA-MB-231 BR “brain-seeking” (231-BR) cell line expressing enhanced green fluorescent protein (eGFP) was produced and kindly provided by Dr. Patricia S Steeg (National Cancer Institute, Bethesda, MD). The cells were maintained in RPMI1640 (Cellgro, Mediatech, Inc., Manassas, VA) supplemented with 10 % fetal bovine serum (Gibco, Invitrogen, Grand Island, NY) and 1 % antibiotic–antimycotic (Invitrogen, Carlsbad, CA). Cells were kept in a humidified atmosphere with 5 % CO_2_ at 37 °C. eGFP expression in the cells was confirmed by fluorescent microscopy. Prior to beginning, the study protocol was approved by the Institutional Animal Care and Use Committee of the Indiana University School of Medicine. All experiments were conducted in accordance with the NIH guidelines for the care and use of laboratory animals. Six- to eight-week-old athymic *nu/nu* female mice (Harlan Sprague–Dawley Inc., Indianapolis, IN) were used to establish human breast metastatic tumor xenografts in the brain. A cell suspension of eGFP expressing 231-BR cell line (231-BR-eGFP) in the exponential growth phase was prepared in a concentration of 250,000 cells in 100 μl HBSS. Under isoflurane anesthesia, the cells were inoculated into the left cardiac ventricle of three mice. After 4 weeks, the mice evidenced symptoms of brain metastasis, e.g., hind limb paralysis. Mice were sacrificed; brains were removed and fixed in 10 % buffered formalin (Protocol™, Fisher Scientific, Kalamazoo, MI). Following paraffin-embedding, 5-μm sections were cut and the sections viewed using an Olympus BX41 fluorescence microscope fitted with a ×40 FITC filter.

A second group of 12 mice underwent intracardiac injection with the 231-BR-eGFP cells as above. Upon evidence of cerebral metastasis, monocytes were isolated and loaded with red fluorescent microspheres as follows: 20.8 × 10^7^ monocytes in 25 mL of RPMI media (Cellgro, Mediatech, Inc., Manassas, VA) supplemented with 10 % fetal bovine serum (Gibco, Invitrogen, Grand Island, NY) and 1 % antibiotic–antimycotic (Invitrogen, Carlsbad, CA) were incubated with 50 μl of red fluorescent FluoSpheres^®^ NeutrAvidin^®^-labeled microspheres (0.04 μm; excitation 580 nm, emission 605 nm; Molecular Probes, Invitrogen, Carlsbad, CA) in a VueLife^®^ FEP cell culture bag (American Fluoroseal Corporation, Gaithersburg, MD) overnight at 37 °C, 5 % CO_2_. To remove any unphagocytosed red fluorescent microspheres, cells were washed with PBS twice followed by centrifugation. A total of 4.1 × 10^7^ cells were collected. The presence of the red fluorescent microspheres in the cells was confirmed by fluorescence microscopy. The cell concentration was adjusted to 10^7^/100 μl by adding 410 μl PBS to the cell pellet. One hundred microliters of the cell suspension was injected into the systemic circulation via the tail vein; 24 h after injection of the monocytes/macrophages, the mice were euthanized.

### Biodistribution analysis of mouse organs following injection of monocytes/macrophages loaded with red fluorescent microspheres

Following the systemic injection of monocytes/macrophages containing the fluorescently labeled microspheres, mice were sacrificed and the brain, lung, liver, and the heart were dissected. The presence and location of metastatic tumor foci (green fluorescence) and monocytes/macrophages loaded with the red fluorescent microspheres in these organs was determined using an optical imaging system, Berthold NightOwl Optical Imager (Berthold Technologies, Oak Ridge, TN) loaded with Berthold Winlight 32 software (Berthold Technologies). The images of green fluorescence and red fluorescence were taken after exposure times of 100 and 500 ms, respectively. The intensity of the fluorescence in different area of the organs was measured using the signal intensity bar.

### Immunofluorescence analysis of monocytes/macrophages in the green fluorescent brain metastatic tumor cells

After imaging, brains were bisected along the sagittal plane, and fixed by perfusion in 4 % paraformaldehyde overnight at 4 °C. For further fixation the brain hemispheres were transferred to 20 % sucrose solution and fixed overnight at 4 °C. The fixed brain was frozen in Tissue-Tek O.C.T. compound (Sakura Finetec, Torrance, CA). Brains were sectioned serially in 5-μm increments, processed for immunofluorescent staining and imaging. eGFP expressing tumor cells in brain metastases and red fluorescent microspheres were detected by confocal microscopy using Olympus FV1000 MPE (Olympus, Center Valley, PA). In order to determine if the red fluorescent microspheres were contained within human monocytes/macrophages, brain sections were stained with anti-human CD68 antibodies (clone C-18; Santa Cruz Biotechnology, Santa Cruz, CA) labeled with APEX Pacific Blue fluorochrome. The labeling of the antibodies was performed using APEX Pacific Blue™ Antibody labeling Kit (Molecular Probes, Invitrogen, Carlsbad, CA) according to the manufacturer’s instruction.

## Results and discussion

**Fig. 1:**
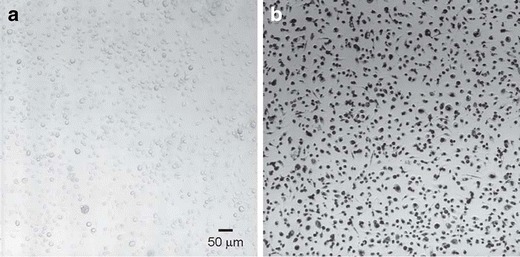
Phagocytosis of gold-silica nanoshells by macrophages. Photomicrographs of transmitted light images of macrophages (control) (**a**) and following 3 days incubation with nanoshells (**b**). The visualization was performed using a Bio-Rad Radiance 2100 MP Rainbow Confocal/Multiphoton System. The images were acquired using the ×10 objective

This study was designed as a pilot to test whether loaded monocytes/macrophages are able to maneuver around the BBB and actively deliver nanoparticles to intracranial metastasis with the potential, to be realized, to deliver effective therapy. In a previous publication, the efficient phagocytosis of gold nanoshells by monocytes/macrophages was demonstrated (Choi et al. 2007). Phagocytosis of nanoshells is so avid, in fact, that the monocytes/macrophages are rendered black in optical imaging (Fig. 1). This appears to occur without exception in every monocyte/macrophage in culture.

**Fig. 2:**
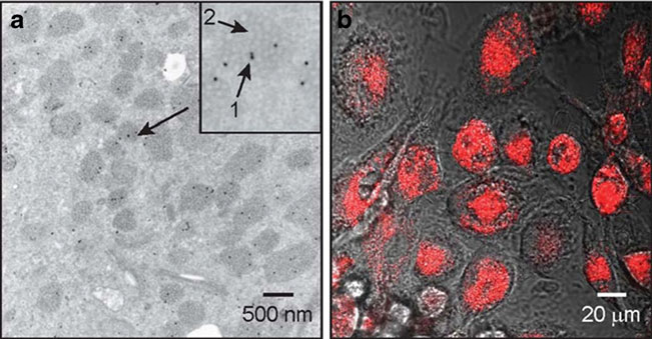
Cellular uptake of gold-silica nanoshells and red fluorescent microspheres by monocytes/macrophages. **a** Transmission electron micrograph of monocytes/macrophages incubated with nanoparticles reveals intracellular localization of gold conjugates (*arrow 1 in inset*) as well as the ingested red fluorescent microspheres located in the vacuoles in the cytoplasm (*arrow 2 in inset*). **b** Fluorescence microscopy imaging shows significant intensity of red fluorescence (580/605 nm) indicating the presence of intracellular red fluorescent microspheres

To detect the presence of the nanoparticle-laden macrophages in the brain, fluorescently labeled microspheres were used to track macrophage location. Although fluorescent nanoshells have been demonstrated that combine both diagnostic and therapeutic functions in the same nanoparticle,(Bardhan et al. 2008, 2009a, 2009b; Tam et al. 2007) here we chose to investigate delivery by a “cocktail” of two different types of nanoparticles, one therapeutic and one contrast agent. Prior to commencing the in vivo experiment, it was determined if monocytes would phagocytose the fluorescently labeled microspheres as avidly as gold nanoshells. Avid uptake of the fluorescently labeled microspheres was observed, as shown in Fig. 2. Although it appears from this two-dimensional photomicrograph that the uptake is within the nucleus, with three-dimensional imaging it can be observed that the microspheres are perinuclear, which is confirmed by transmission electron microscopy (TEM) imaging. The TEM image also shows a much larger number of microspheres phagocytosed in relation to the numbers of gold conjugate. As with the phagocytosis of the nanoshells, the phagocytosis of the microspheres and gold conjugates appears to be robust in every monocyte/macrophage exposed to the nanoparticles.

**Fig. 3:**
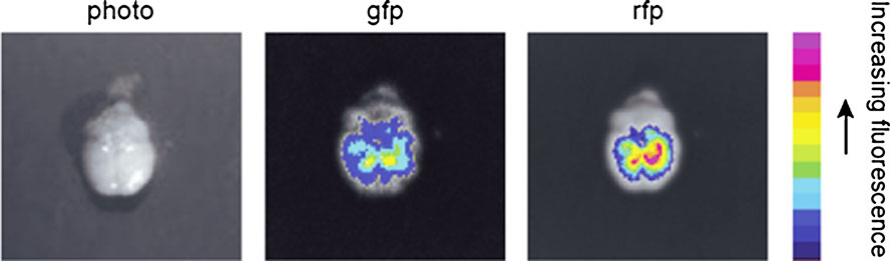
Ex vivo images of whole brain of mice injected with monocytes/macrophages loaded with red fluorescent microspheres. The presence and location of metastatic brain tumor foci (green fluorescence) and macrophages loaded with the red fluorescent microspheres was determined after resection using an optical imaging system. The images of green fluorescence (green fluorescent protein, *gfp*) and red fluorescence (red fluorescent particles, *rfp*) were taken after exposure times of 100 and 500 ms, respectively. The intensity of the fluorescence in different area of the brain is measured using the signal intensity bar. Values are normalized relative to the exposure times

To test the hypothesis that systemically injected, nanoparticle-laden monocytes/macrophages would home in to intracranial metastatic deposits by crossing the blood–brain barrier a mouse model of breast cancer metastatic to the brain, the human MDA-MB-231 BR “brain-seeking” (231-BR) cell lines expressing enhanced green fluorescent protein (eGFP), was utilized (Yoneda et al. 2001; Palmieri et al. 2007). Twelve mice underwent intracardiac injection of the 231-BR cells of which three died following injection. The remaining nine mice underwent tail vein injection of the nano-laden macrophages; at necropsy it was determined that six of these nine mice had actually developed brain metastasis, three had not. Imaging of the freshly dissected brains of mice injected 24 h earlier with the loaded macrophages reveals that the green [metastasis] and red [microspheres] fluorescence overlap each other with maximal intensity in a posterior direction (Fig. 3). This suggests that the microspheres have been successfully delivered to the metastases.

**Fig. 4:**
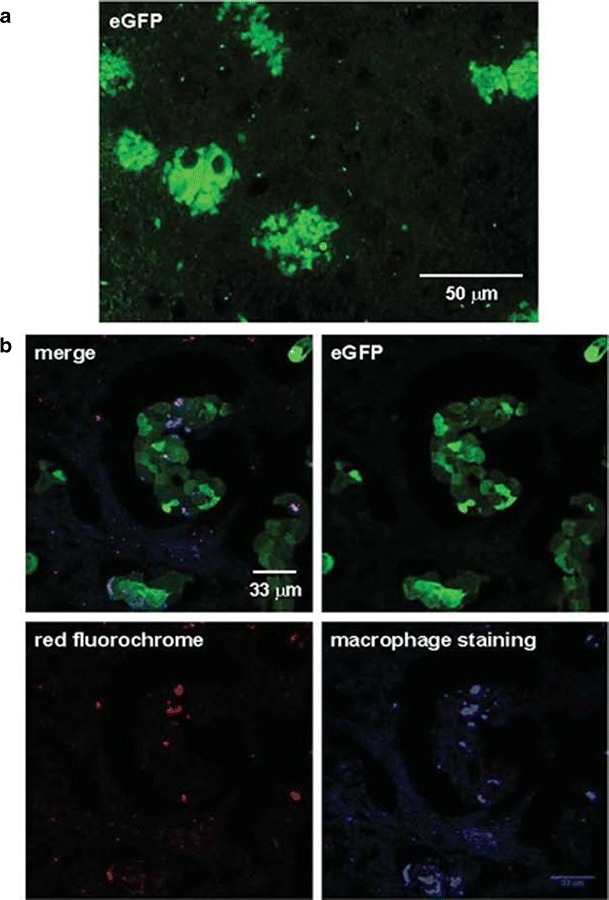
Histologic images of brain metastatic tumor cells. **a** Paraffin-embedded section of the brain of a control mouse showing the formation of metastatic brain lesions derived from eGFP expressing breast cancer cells. **b** Macrophages loaded with red fluorescent nanospheres infiltrating the brain metastases detected by immunofluorescent staining using anti-human CD68 antibodies labeled with APEX Pacific Blue fluorochrome. *Green fluorescence* is emitted by the brain metastatic tumor cells. *Red fluorescence* and *blue fluorescence* result from the red fluorescent nanospheres included within the macrophages and the macrophage staining, respectively

Confocal microscopy of the brain sections reveals eGFP expressing tumor cells forming brain metastases (Fig. 4a). Red fluorescence from the microspheres was observed within the metastases of two of the six mice with brain metastases. To determine if the red fluorescent microspheres were contained within human macrophages, brain sections were stained with anti-human CD68 antibodies labeled with APEX Pacific Blue fluorochrome. As observable in Fig. 4b, the microspheres are contained with the macrophages, which are intercalated within the metastases. Tail vein injection in athymic *nu/nu* mice is technically challenging given the small size of this vessel. In the four mice for which no delivery of nanoparticles is observed, the hypothesis is that the needle transversed the vein and the macrophage suspension infiltrated the subcutaneous tissue surrounding the tail vein rather than entering it and the systemic circulation.

**Fig. 5:**
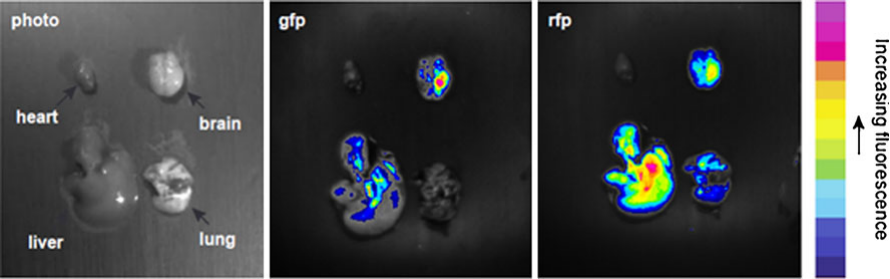
Biodistribution images of mouse organs following injection of monocytes/macrophages loaded with red fluorescent nanospheres. The distribution of metastatic tumor foci (*green fluorescence*) and macrophages loaded with the red fluorescent nanospheres in the brain, lung, liver, and the heart was determined after resection using an optical imaging system. The images of *green fluorescence* (green fluorescent protein, *gfp*) and red fluorescence (red fluorescent particles, *rfp*) were taken after exposure time of 600 ms. The intensity of the fluorescence in different area of the organs is measured using the signal intensity bar. There is abundant uptake of microsphere-laden macrophages in the liver. Brain metastases are located in the left hemisphere of the brain. The distribution of the *red fluorescence* within the brain overlaps that of the green fluorescence suggesting that the microsphere-laden macrophages are co-located with the cerebral metastases

Whole-body fluorescence imaging of mice following the systemic injection of macrophages containing the fluorescently labeled microspheres shows an initial [within minutes] and temporary residence in the lungs followed by residence in the liver and spleen which occurs over hours to days (Fig. 5 and data not shown).

Our work is focused on the treatment of metastases from breast cancer. This is a critical unmet clinical need. The purpose our study was to test the hypothesis that nanoparticle-laden monocytes/macrophages would home in to intracranial metastatic deposits by crossing the blood–brain barrier following injection into the systemic circulation. The activated macrophages not only cross the BBB but as shown in Fig. 3, they envelop the metastatic cells delivering the loaded nanoparticle to less than a cell width away from the nearest metastatic cell. This “Trojan Horse” delivery method has been designed to address the challenges to successful cancer nanotherapeutics posed by the tumor microenvironment. It takes advantage of the recruitment of macrophages to the metastatic lesions; this is active transport of the nanovectors likely the result of the productions of chemoattractant(s) by the metastasis, which does not require the Enhanced Permeability and Retention effect or diffusion of the nanovector.

Injection of monocytes/macrophages into the systemic circulation resulted in residence in the liver and lungs as well as transport to the brain metastasis. This result was predictable. Previous studies have demonstrated that when marrow derived mononuclear cells or peritoneal macrophages are injected into the systemic circulation they home to the same tissues and have a distribution similar to endogenous macrophages (Kennedy and Abkowitz 1998; Freeman et al. 1999). This distribution is to treatment advantage. Monocyte/macrophages as delivery vehicles can be envisioned as a pan-metastatic treatment because, in addition to the brain, the lungs and liver are frequent sites of metastatic disease. What is unknown and to be determined in follow-on studies is the exact percentage of the administered dose that actually crosses the BBB. Also to be determined is the rate at which the macrophage will discharge its cargo and whether or not it will be necessary to destroy the macrophage for this to happen.

## Conclusions

To our knowledge, we report the first successful demonstration of the active delivery, using macrophages, of nanoparticles to brain metastases and it paves the way for testing this method to deliver other nanovectors including nanorods, hollow nanospheres in the sub-100- and sub-50 nm range as well as nanoformulated therapeutics.
